# Pontin Acts as a Potential Biomarker for Poor Clinical Outcome and Promotes Tumor Invasion in Hilar Cholangiocarcinoma

**DOI:** 10.1155/2018/6135016

**Published:** 2018-05-13

**Authors:** Qi Sun, Fanni Li, Songyang Yu, Xiang Zhang, Feiyu Shi, Junjun She

**Affiliations:** ^1^Department of General Surgery, The First Affiliated Hospital of Xi'an Jiaotong University, Shaanxi, Xi'an 710061, China; ^2^Department of Talent Highland, The First Affiliated Hospital of Xi'an Jiaotong University, Shaanxi, Xi'an 710061, China; ^3^Second Affiliated Hospital of Shandong University, Shandong, Jinan 250033, China; ^4^Department of Urology, Qilu Hospital of Shandong University, Shandong, Jinan 250012, China

## Abstract

Hilar cholangiocarcinoma (HC) is a devastating malignancy that carries a poor overall prognosis. As a member of the AAA+ superfamily, Pontin becomes highly expressed in several malignant tumors, which contributes to tumor progression and influences tumor prognosis. In our research, Pontin expression in tumor specimens resected from 86 HC patients was detected by immunohistochemistry. Interestingly, high expression of Pontin was significantly associated with lymph node metastasis (*p* = 0.011) and tumor node metastasis (TNM) stage (*p* = 0.005). The Kaplan–Meier overall survival rate and multivariate analyses were performed to evaluate the prognosis of patients with HC. Patients with high Pontin expression had significantly poorer overall survival outcomes. Multivariate analyses found that Pontin was an independent prognostic factor (*p* = 0.001). Moreover, bioinformatics analysis confirmed the increase in Pontin mRNA expression levels in cholangiocarcinoma tissues. In addition, in vitro experiments showed that Pontin expression was inhibited at the mRNA as well as protein levels after transfection with Pontin siRNA in human cholangiocarcinoma cell lines. Moreover, significant suppression of cell invasion was observed after the downregulation of Pontin. Taken together, the present study suggested that Pontin could act as a potential prognostic predictor, which might be a new valuable molecular candidate for the prevention and treatment of HC.

## 1. Introduction

Hilar cholangiocarcinoma (HC), or Klatskin's tumor, was first described in 1965 and is a devastating malignant disease with high rates of metastasis [1]. Both liver resection and orthotopic liver transplantation offer the potential chance of a cure, while adjuvant therapy contributes little to the overall survival (OS) of HC cases [2–4]. HC is often diagnosed at a late stage when the regional and para-aortic lymph nodes are frequently involved and the majority of patients have lost the chance of complete resection [5, 6]. A better understanding of the underlying biological mechanisms might help to better identify patients with good prognosis as well as high-risk patients and to improve the efficacy of additional treatment options in HC; therefore, exploring the novel prognostic markers of this specific tumor entity is urgently needed.

Pontin, also named as Tip49a or RUVBL1, which is closely related to the bacterial RuvB DNA helicases, belongs to the AAA+ family of ATPases that share remarkable conservation from yeast to humans [7]. The Pontin gene is located on chromosome 3q21, which encodes a 456-amino acid protein with a molecular weight of 52 kDa [8]. Pontin is an intriguing protein that seems to be implicated in processes including the regulation of gene transcription, remodeling of chromatin, DNA damage sensing and repair, and assembly of protein and ribonucleoprotein complexes. More notably, Pontin was found to be overexpressed in many cancer types and was shown to play an important role in tumor biology [9]. The first hint of a relationship between Pontin and cancer came from the discovery that Pontin was implicated in the stimulation or repression of certain oncogenic transcriptional factors, including *β*-catenin and c-Myc [8, 10]. Furthermore, Pontin and its related partner Reptin (RUVBL2, TIP48, and Tip49b) share residence in several multiprotein complexes such as the Tip60, INO80, SRCAP, and Uri complexes [11], which suggests that these complexes modulate transcriptional programs in several different cellular and molecular contexts [12, 13]. Aberrant Pontin expression has been reported in liver cancer [14], colon carcinoma [15], renal cell carcinoma (RCC) [16], and acute myeloid leukemia (AML) [17]. However, the expression and clinicopathologic characteristics of Pontin in HC have not been investigated to date, and it remains uncertain whether Pontin is of importance to the poor survival of HC patients.

In the present study, for the first time, we examined the expression level of Pontin in HC surgical samples by immunohistochemistry and attempted to evaluate its clinicopathological association and prognostic implications. Our data indicated that Pontin is crucial in the malignant progress of HC. Furthermore, the expression of Pontin protein was detected by Western blot in 16 pairs of fresh frozen HC samples and matched adjacent normal bile duct tissues. Additionally, we analyzed the Pontin mRNA level in cholangiocarcinoma tissues compared with the nontumor-surrounding tissues by GSE26566 from the GEO database. Furthermore, we demonstrated that Pontin suppression could significantly suppress the invasive capability of both RBE and QBC939 cell lines in vitro. These results may provide a novel strategy for the development of targeted therapy for HC.

## 2. Materials and Methods

### 2.1. Clinical Samples

For Western blot analysis, 16 pairs of pathologically confirmed HC tissues and matched adjacent normal bile duct tissues were obtained from patients undergoing curative surgical resection of HC from October 2013 to May 2016 at Qilu Hospital of Shandong University. Eighty-six patients diagnosed with HC who received curative surgery were compiled consecutively between January 2006 and December 2015 at Qilu Hospital of Shandong University. There were 54 males and 32 females, with the median age of 56 years old (range: 37–79 years). The medical records of these patients were reviewed to extract clinical and histopathological information. All patients were followed up until December 2016. The median follow-up period was 20 months (range: 5–120 months). Patients who do not have the intact follow-up data were ruled out from the present study. Pathologic tumor node metastasis (pTNM) classification was based on the American Joint Committee on Cancer (AJCC)/Union for International Cancer Control (UICC, 7th edition, 2010). The experimental protocols were approved by the ethics committee of Qilu Hospital of Shandong University and informed consent had been obtained from all patients.

### 2.2. Immunohistochemistry

Representative formalin-fixed, paraffin-embedded tissues were retrieved from Qilu Hospital of Shandong University. All the samples were sectioned into 5 *μ*m thick slides in succession, which were subjected to routine deparaffinization, rehydration, and optimal antigen retrieval performed in citrate buffer (pH 6.0) at 98°C for 15 min. The slides were incubated in 5% hydrogen peroxide for 20 min and then were blocked with 10% normal goat serum for 30 min. The slides were incubated with primary anti-Pontin antibody (1 : 100, Abcam, Cambridge, MA, USA; Cat. number ab51500) overnight at 4°C and were then incubated with HRP-conjugated anti-mouse IgG. The immunostaining was achieved with DAB (Beyotime, Shanghai, China). Finally, the sections were washed and counterstained with hematoxylin. The immunostaining was evaluated, respectively, by two independent observers.

### 2.3. Cell Culture and Transfection

The human cholangiocarcinoma cell lines RBE and QBC939 (ATCC, USA) were separately maintained in RPMI 1640 and DMEM medium (Gibco Co., USA), respectively, supplemented with 10% fetal bovine serum (Gibco Co., USA) in a humidified incubator with 5% CO_2_. Both RBE and QBC939 cells were transfected with Pontin siRNA using Lipofectamine 2000 according to the manufacturer's protocols (Invitrogen, Carlsbad, CA). The sequence of Pontin siRNA was as follows: 5′-CCA UGC UGU AUA CUC CAC AGG AAA U-3′. The negative control siRNA (si-NC) were synthesized by GenePharma (Biosune, Shanghai China).

### 2.4. RNA Isolation and Quantitative Real-Time PCR (qRT-PCR)

Total RNA was extracted from cells by E.Z.N.A.® Total RNA Kit II (Omega Bio-Tek, Norcross, GA). The reverse transcription reaction was performed using ReverTra Ace Kit (Toyobo, Osaka, Japan). SYBR-Green real-time PCR was analyzed using the Stratagene Mx3005P sequence detection system (Agilent Technologies, Santa Clara, CA) with SYBR Premix EX Taq II (2x) (Takara, Shiga, Japan). The relative mean fold change in the expression ratios was calculated by the 2^−ΔΔCt^ method. The primers for human Pontin were as follows: 5′-GGCATGTGGCGTCATAG TAGA-3′ (forward) and 5′-CACGGAGTTAGCTCTGTGACT-3′ (reverse). Expression data were uniformly normalized to *β*-actin as the internal control. The primers of *β*-actin were as follows: 5′-GGGACCTGACTGACTACCTC-3′ (forward) and 5′-TCATACTCCTGCTTGCTGAT-3′ (reverse).

### 2.5. Assessment of Pontin Immunostaining

Pontin immunostaining was observed primarily in the cytoplasm as well as nuclei of the tumor cells. Pontin staining was assessed based on both the intensity and extent of immunostaining using a semiquantitative method. In brief, the intensity of staining was scored as 0 (low), 1 (weak), 2 (medium), or 3 (strong), and the extent of staining was scored as 0 (0–5%), 1 (6–25%), 2 (26–50%), 3 (51–75%), or 4 (76–100%). The summed score of the intensity and extent was used as the final immunohistochemistry score and ranged from 0 to 7.

Because the immunohistochemistry scores of Pontin immunostaining did not show a biphasic distribution, the optimal cutoff point was determined according to the heterogeneity value, which was measured using the log-rank test [18]. In brief, every immunostaining score ranging from 1 to 6 was used as the cutoff point at which the patients were divided into two groups, and then the log-rank test was used to analyze the difference between these groups with respect to the OS. Finally, we found that the optimal cutoff point was at the immunostaining score of 5, which was chosen to discriminate between low and high Pontin expression.

### 2.6. Western Blot

Frozen tissue samples were lysed on ice in RIPA buffer (Cell Signaling Technology, Danvers, USA) containing complete protease inhibitor cocktail (Roche Applied Science, Mannheim, Germany). Equal amounts of protein were separated on SDS-PAGE and were transferred to polyvinylidene fluoride membranes (Sigma-Aldrich; Merck KGaA, Darmstadt, Germany). The membranes were blocked for 1 h at room temperature in 5% nonfat dry milk in TBST buffer and were then incubated overnight with the primary antibodies anti-Pontin (1 : 100; Abcam) or anti-*β*-actin (1 : 1000, Santa Cruz Biotechnologies, USA). Following washing with TBST three times at room temperature, the membranes were incubated with horseradish peroxidase-conjugated secondary antibodies for 1 h. The membranes were visualized by chemiluminescence (Millipore, USA).

### 2.7. Transwell Assay for Invasion

Tumor cell invasion assay was analyzed in 24-well plate containing 8 *μ*m pore size of transwell chamber (Costar, Acton, MA) precoated with 50 *μ*l of Matrigel (BD Bioscience, San Jose, CA). Pontin siRNA was transfected into the human cholangiocarcinoma cell lines RBE and QBC939. After transfection for 48 h, the cells were trypsinized, and 1 × 10^5^ cells were seeded to the upper chamber in 200 *μ*l RPMI 1640 or DMEM serum-free medium. The lower chamber was filled with 700 *μ*l of RPMI 1640 or DMEM medium containing 10% FBS as chemoattractants. After incubation for 24 h, a cotton swab was used to remove the noninvaded cells on the upper membrane surface, which were fixed with methanol and then stained with 0.1% gentian violet. The stained cells were counted in at least five high-power fields (×400) under a light microscope for the assessment of the invaded cells.

### 2.8. Bioinformatics

The mRNA expression level of Pontin between the nontumor-surrounding liver and cholangiocarcinoma tissues was measured by GSE26566, which is available in the GEO database (https://www.ncbi.nlm.nih.gov/geo/query/acc.cgi?acc=GSE26566) [19].

### 2.9. Statistical Analyses

SPSS 18.0 software was used for statistical analysis. Chi-square test and Fisher's exact test were used to analyze the relationship between Pontin expression and clinicopathological factors. Overall survival analysis was performed using the Kaplan–Meier estimates and log-rank tests. Cox analyses were used to evaluate Pontin expression as well as other prognostic factors with respect to OS. Student's *t*-test was used to compare two independent groups of data; *p* < 0.05 was considered to indicate a statistically significant difference.

## 3. Results

### 3.1. Expression of Pontin in HC

**Figure 1:**
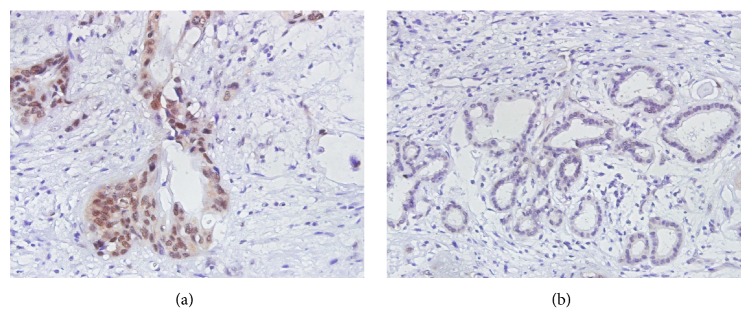
Immunohistochemical results of Pontin in human hilar cholangiocarcinoma tissues (400x). (a) High Pontin expression (score = 6); (b) low Pontin expression (score = 0).

Representative images of Pontin immunostaining are shown in Figure 1. We found that Pontin expression was observed mainly in the cytoplasm and nuclei of tumor cells (Figure 1). Using an optimal cutoff point of 5, patients with HC were separated into low Pontin expression (scores < 5) and high Pontin expression (scores ≥ 5) groups irrespective of other factors (e.g., age, gender, and TNM). In the 86 carcinoma specimens, high Pontin expression was found in 34 samples (39.5%), while low Pontin expression was found in 52 samples (60.5%).

### 3.2. Association between Pontin Expression and Clinicopathological Characteristics in Patients with HC

To further dissect the role of Pontin in HC, we evaluated its potential correlation with demographic factors (gender, age) and clinicopathological parameters in patients with HC. The relationships between Pontin expression and clinicopathological characteristics of these patients are summarized in Table 1. High Pontin expression was closely related to lymph node metastasis (*p* = 0.011) and TNM stage (*p* = 0.005). However, the analysis did not reveal any significant association between high and low Pontin expression with respect to age, gender, tumor differentiation, Bismuth–Corlette classification, or tumor size.

### 3.3. Prognostic Value of Pontin Expression in HC

**Figure 2:**
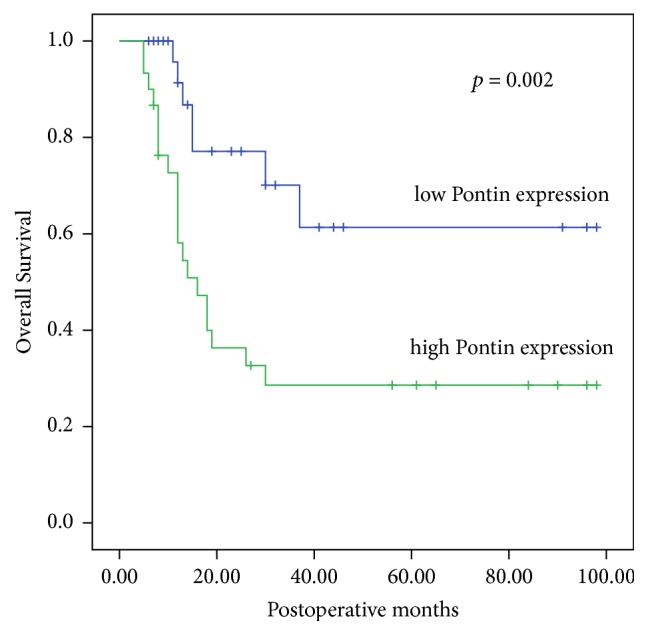
Overall survival curves of patients with hilar cholangiocarcinoma with respect to different Pontin expression. Patients with high Pontin expression had significantly poorer OS than those with low Pontin expression (*p* = 0.002).

Kaplan–Meier analysis of OS was used to indicate the association between Pontin expression and patient survival. As shown in Figure 2, patients with high Pontin expression had a poorer OS time than those with low Pontin expression (*p* = 0.002). Univariate analysis by the log-rank test revealed that the clinicopathological characteristics, including differentiation, lymph node metastasis, TNM stage, and Pontin expression, were significantly associated with OS, while other features were not (Table 2). Furthermore, a Cox proportional hazard regression model was performed to establish the independent prognostic factors. Multivariate analyses showed that only high Pontin expression (*p* = 0.001) and lymph node metastasis (*p* = 0.005) were confirmed as independent prognostic indicators for HC patients, suggesting that high Pontin expression is a high-risk factor for poor prognosis (Table 3).

### 3.4. Enhanced Expression of Pontin in HC

**Figure 3:**
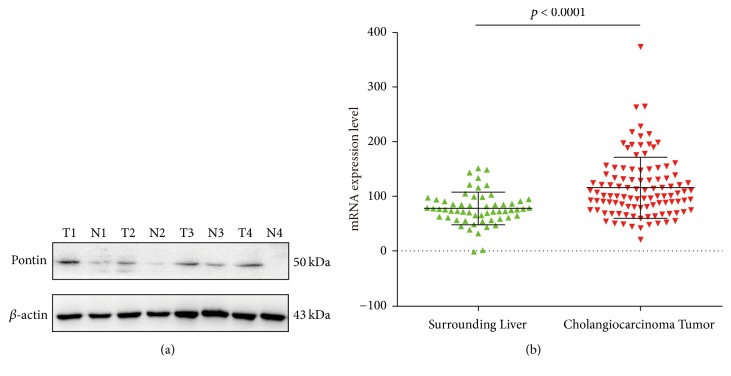
Pontin protein expression in HC tissues and bioinformatics analysis of the Pontin mRNA levels from the GEO database. (a) Western blot analyses of 4 representative pairs of HC tissues (T) and adjacent normal bile duct tissues (N). *β*-actin was used as a loading control. (b) GEO data indicated significantly higher Pontin mRNA expression in cholangiocarcinoma tissues than in nontumor-surrounding liver tissues.

Western blotting was carried out to detect the expression of Pontin protein levels in the 16 paired fresh frozen HC tissues and the matched adjacent normal bile duct tissues. Among the 16 pairs of HC tissues and matched adjacent normal bile duct tissues, there were 12 pairs which demonstrated significantly increased Pontin expression in HC tissues, and for the rest 4 pairs, the difference of Pontin expression was not so significant (Supplemental Table 1). As shown in Figure 3(a), the Pontin protein level was significantly increased in HC tissues compared with in matched normal bile duct tissues. We next utilized the GEO database to explore the differences in the Pontin mRNA expression between the nontumor-surrounding liver and cholangiocarcinoma tissues. The analysis revealed that the Pontin mRNA level was significantly differentially expressed between tumor and nontumor-surrounding liver tissues (*p* < 0.0001, Figure 3(b)).

### 3.5. Role of Pontin in Cell Invasion

**Figure 4:**
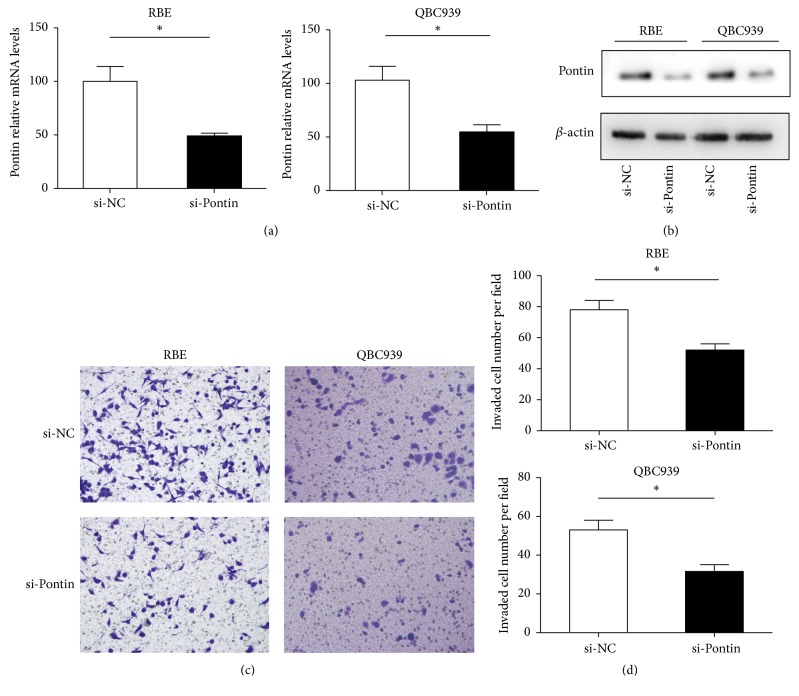
Role of Pontin in cholangiocarcinoma cell invasion. (a) Effects of Pontin siRNA on Pontin mRNA levels as detected through qRT-PCR. (b) Effects of Pontin siRNA on Pontin protein levels as detected through Western blot. ((c) and (d)) The invasive capacity of RBE and QBC939 cells was evaluated by the Matrigel invasion assay. The data are shown as the means ± SEM. (*n* = 3) ^*∗*^*p* < 0.05.

The mRNA and protein expression levels of Pontin were significantly decreased in RBE and QBC939 cells through Pontin siRNA (Figures 4(a) and 4(b)). To analyze the effects of Pontin inhibition on cell invasion, the transwell-Matrigel invasion assay was performed. Invading cells was quantified by staining, and the results revealed that Pontin depletion in RBE and QBC939 cells significantly restrained the invasive ability of cells (Figures 4(c) and 4(d)). The potential effect of cell proliferation on cell invasion was rolled out (Supplemental Figure 1).

## 4. Discussion

As a complex and aggressive tumor, HC is notorious for its extremely poor prognosis. Lymph node metastasis and disease stage are the most accepted prognostic factors for HC. In addition, the presence of tumor markers is related to disease progression and OS. However, few of these biomarkers have successfully been applied to clinical practice. To prolong the OS of HC patients, it is necessary to identify novel biomarkers to monitor the progression of HC and to develop effective therapeutic targets for HC.

Increasing evidence has suggested that the AAA+ superfamily member Pontin, as a homology partner of Reptin, is highly relevant to some progressive malignancies, including colorectal, hepatocellular, prostate, and nonsmall cell lung cancers. The clinical significance of Pontin, to the best of our knowledge, has not been studied in HC. This is the first report to analyze the association of Pontin expression with the clinical significance in HC.

In the present study, we investigated Pontin expression in patients with HC by immunohistochemistry and examined the relationships between Pontin expression levels and clinicopathological factors and patient outcomes. Unexpectedly, the immunohistochemical results demonstrated that high Pontin expression was found in 34 of the 86 HC patients (39.5%). The mechanism underlying the increased Pontin expression in cancer remains controversial and may be tumor-type-specific. There is little evidence that Pontin overexpression in cancer is consecutive to gene amplification. A previous study showed that two single-nucleotide polymorphisms were identified in the Pontin gene that were related to an increased risk of ovarian carcinoma [20]. These polymorphisms may be the cause of the variations in the expression of Pontin. Therefore, further investigations are required regarding the molecular mechanisms of Pontin overexpression in HC.

We also compared the expression of Pontin among different clinicopathological factors, including tumor stage, lymph node metastasis, and TNM stage, to test whether Pontin was related to certain clinical characteristics. Pontin was strongly associated with the aggressiveness of HC. The presence of lymph node metastasis, recognized as the most common metastatic lesion, is considered an important prognostic factor for patients with HC. In addition to lymph node metastasis, the TNM stage is a recognized parameter for predicting the prognosis of malignant tumors. In this study, lymph node metastasis (*p* = 0.011) and advanced TNM stage (*p* = 0.005) were related to high Pontin expression, which was consistent with previous reports. Haurie et al. [21] reported the correlation of high Pontin expression with poor prognosis in HCC patients. Lauscher et al. [15] found that the upregulation and nuclear localization of Pontin and *β*-catenin may help promote the progression of colon cancer. In our study, the level of Pontin was not related to gender (*p* = 0.451), age (*p* = 0.329), or Bismuth–Corlette classification (*p* = 0.624). Therefore, high expression of Pontin was correlated significantly with disease progression, including tumor invasion and lymph node metastasis. Kaplan–Meier analysis indicated a significantly lower OS rate in patients with high Pontin expression than in those with low Pontin expression. Moreover, high Pontin expression was an independent prognostic indicator for poor survival outcomes of HC, which was confirmed by the multivariate Cox proportional hazards model. In addition, we measured the mRNA levels of Pontin between cholangiocarcinoma and nontumor-surrounding normal tissues using a bioinformatics database, and we found that Pontin mRNA levels were upregulated in cholangiocarcinoma tissues compared with adjacent normal tissues (*p* < 0.0001); this finding was consistent with those of previous studies reporting that Pontin is highly upregulated in nonsmall cell lung cancer [22]. The transwell invasion assay confirmed that the invasion capacities of both RBE and QBC939 cells were significantly inhibited following Pontin depletion by siRNA. The current findings demonstrate that Pontin may play an important role in the malignancy of HC.

As a highly conserved AAA+ ATPase, Pontin is related to multiple complexes that carry out essential cellular functions. A main feature of Pontin is its participation in various ATP-dependent chromatin-remodeling complexes, including p400, SWR1, INO80, and Tip60. These chromatin-remodeling complexes play a key role in not only transcription but also cell cycle checkpoint activation, centromere stability, telomere regulation, and chromosome segregation [23–25]. A link between Pontin and cancer has been established, as Pontin was found to be overexpressed in many cancers and was shown to play roles in tumor biology [26]. Notably, Pontin interacts directly with the TATA-binding protein and acts as a cofactor for several transcription regulators strongly involved in oncogenic pathways, such as *β*-catenin and c-Myc, both of which were associated with the potential for malignancy in HC [27–29]. Pontin has previously been shown to interact with *β*-catenin and to enhance TCF/*β*-catenin-mediated transcription of Wnt genes; thus, it may contribute to cancer progression, while c-Myc is one of the most frequent sites of mutation in human cancer. Reporter gene assays showed that Pontin could enhance *β*-catenin-TCF transcriptional activity [30]. A previous study reported that the nuclear colocalization of Pontin and *β*-catenin was involved in the progression of colon cancer [15], and Pontin depletion was found to significantly decrease nuclear *β*-catenin in renal cell carcinoma cell lines [16]. In addition, Pontin was shown to directly interact with c-Myc and control its oncogenic activity [29]. Unexpectedly, binding sites for c-Myc were found in the promoter of Pontin; thus, c-Myc could probably regulate Pontin transcription directly [31, 32]. Given that c-Myc is aberrantly activated in various cancers, including HC, this may provide a plausible explanation for the increased levels of Pontin in HC [33].

In our study, we showed that high Pontin expression had a significant prognostic value for patients with HC. High expression of Pontin is associated with poor survival in patients with HC. Interestingly, Havlik et al. reported that p53 mutation was also associated with poor survival in HC [34]. Furthermore, Zhao et al. found that Pontin as a novel mutp53-interacting protein could promote mutp53 gain-of-function in the invasion and anchorage-independent growth of tumor cells by regulating the transcriptional activity of mutp53 toward a subset of its target genes [10]. Additionally, a previous study showed that Pontin could downregulate wtp53 levels and function in human colorectal cancer RKO cells [35]. In this context, Pontin overexpression has biological significance in tumors, especially those containing mutp53. These results raise much interest in targeting the Pontin-mutp53 complex to inhibit the invasion and metastasis of HC in further studies.

Interestingly, Pontin and Reptin may function cooperatively or oppositely depending on the transcription factors with which they associate. For example, on the one hand, either Pontin or Reptin could potentiate the ability of Myc to inhibit Miz1 and repress its target gene p21^CIP1^ [36]. On the other hand, they could function as antagonistic regulators of *β*-catenin signaling activity [30]. In addition, previous studies have shown that Pontin and Reptin are attractive targets for cancer therapy. In this respect, antagonizing the ATPase activity of Pontin appears to be an attractive option and could be feasible, and a small molecule antagonizing the ATPase activity of Pontin has been discovered [37]. Thus, exploring the underlying mechanisms of Pontin and Reptin in tumorigenesis is necessary and might lead to the identification of novel compounds or strategies targeting Pontin for further study.

## 5. Conclusions

In conclusion, the present study suggested that high Pontin expression was related to unfavorable clinicopathological factors and decreased survival in human HC. As Pontin acts as an independent predictor of poor postoperative outcomes, targeting Pontin might indicate new directions for antitumor strategies regarding HC that could be assessed in future studies.

## Figures and Tables

**Table 1:** Relationships between the expression of Pontin and clinicopathological features in human hilar cholangiocarcinoma.

Clinicopathological features	*n*	Pontin		*p*
		Low	High	
Gender				0.451
Male	54	31	23	
Female	32	21	11	
Age (years)				0.329
<60	41	27	14	
≥60	45	25	20	
Differentiation				0.062
Well/moderately	51	35	16	
Poorly	35	17	18	
Bismuth–Corlette classification				0.624
Type I	22	13	9	
Type II	11	9	2	
Type IIIa	12	7	5	
Type IIIb	15	9	6	
Type IV	26	14	12	
Tumor size (cm)				0.299
<3	31	16	15	
3–5	19	14	5	
≥5	36	22	14	
Tumor stage				0.127
T1	20	9	11	
T2	27	20	7	
T3	39	23	16	
Lymph node metastasis				0.011^*∗*^
No	59	41	18	
Yes	27	11	16	
TNM stage				0.005^*∗∗*^
I	23	19	4	
II	20	15	5	
IIIa	16	7	9	
IIIb	27	11	16	

^*∗*^ *p* < 0.05; ^*∗∗*^*p* < 0.01.

**Table 2:** Univariate analysis of clinicopathological features for OS of 86 patients with hilar cholangiocarcinoma.

Characteristics	*n*	Survival rate (%)	*p*
Gender			0.544
Male	54	38.9	
Female	32	46.9	
Age (years)			0.497
<60	41	46.3	
≥60	45	37.8	
Differentiation			0.021^*∗*^
Well/moderately	51	52.9	
Poorly	35	25.7	
Bismuth–Corlette classification			0.357
Type I	22	40.9	
Type II	11	45.5	
Type IIIa	12	41.7	
Type IIIb	15	46.7	
Type IV	26	38.5	
Tumor size (cm)			0.299
<3	31	41.9	
3–5	19	47.3	
≥5	36	38.9	
Tumor stage			0.316
T1	20	45.0	
T2	27	48.1	
T3	39	35.9	
Lymph node metastasis			0.011^*∗*^
No	59	47.5	
Yes	27	29.6	
TNM stage			0.044^*∗*^
I	23	56.5	
II	20	45.0	
IIIa	16	31.2	
IIIb	27	29.6	
Pontin expression			0.002^*∗∗*^
Low	52	50.0	
High	34	29.4	

^*∗*^ *p* < 0.05; ^*∗∗*^*p* < 0.01.

**Table 3:** Multivariate analysis of clinicopathological features for OS of 86 patients with hilar cholangiocarcinoma.

Factors	Category	*p*	HR	95% CI
Pontin expression	Low	0.001^*∗∗*^	2.883	1.257–6.724
	High			

Tumor differentiation	Well/moderately	0.592	0.786	0.415–3.539
	Poorly			

Lymph node metastasis	No	0.005^*∗∗*^	2.105	1.101–4.264
	Yes			

TNM stage	I	0.362	4.632	0.826–11.371
	II			
	IIIa			
	IIIb			

^*∗∗*^ *p* < 0.01; HR: hazard ratio; CI: confidence interval.

## Data Availability

All relevant data are within the paper.

## Supplementary Materials

Supplementary Materials Supplemental Table 1: the fold of change of Pontin expression (Pontin/*β*-actin) in all the 16 pairs of HC tissues and matched adjacent normal bile duct tissues. Supplemental Figure 1: effects of Pontin on cholangiocarcinoma cell proliferation in 24-hour serum-free medium culture. MTT assay showed that there were no significant differences in proliferation before and after transfection of si-Pontin to RBE (A) and QBC939 (B) cells.
