# Supplementary material for: Pontin Acts as a Potential Biomarker for Poor Clinical Outcome and Promotes Tumor Invasion in Hilar Cholangiocarcinoma
Source: Biomed Res Int. 2018 May 13;2018:6135016. doi: 10.1155/2018/6135016 (PMC5971300; doi:10.1155/2018/6135016)
Supplement: Supplementary Materials — Supplemental Table 1: the fold of change of Pontin expression (Pontin/β-actin) in all the 16 pairs of HC tissues and matched adjacent normal bile duct tissues. Supplemental Figure 1: effects of Pontin on cholangiocarcinoma cell proliferation in 24-hour serum-free medium culture. MTT assay showed that there were no significant differences in proliferation before and after transfection of si-Pontin to RBE (A) and QBC939 (B) cells. [file 6135016.f1.zip › Supplemental Figure 1_BMRI_2245887.docx]

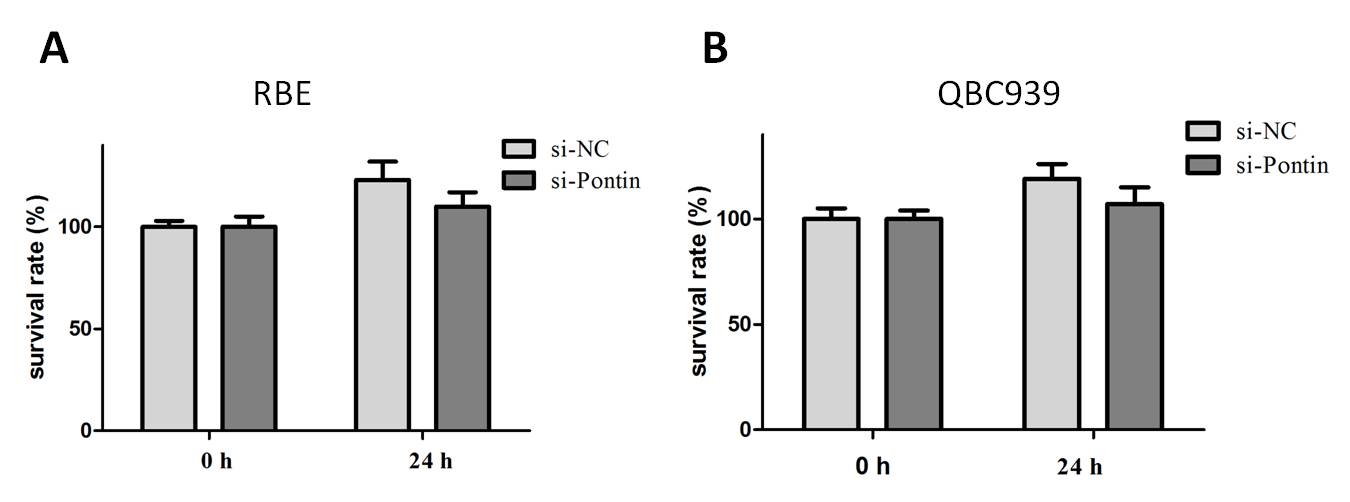


**Supplemental Figure 1**. Effects of Pontin on cholangiocarcinoma cell proliferation in 24-hour serum-free medium culture. MTT assay showed there were no signiﬁcant differences in proliferation before and after transfection of si-Pontin to RBE (A) and QBC939 (B) cells.
