# Supplementary material for: Pontin Acts as a Potential Biomarker for Poor Clinical Outcome and Promotes Tumor Invasion in Hilar Cholangiocarcinoma
Source: Biomed Res Int. 2018 May 13;2018:6135016. doi: 10.1155/2018/6135016 (PMC5971300; doi:10.1155/2018/6135016)
Supplement: Supplementary Materials — Supplemental Table 1: the fold of change of Pontin expression (Pontin/β-actin) in all the 16 pairs of HC tissues and matched adjacent normal bile duct tissues. Supplemental Figure 1: effects of Pontin on cholangiocarcinoma cell proliferation in 24-hour serum-free medium culture. MTT assay showed that there were no significant differences in proliferation before and after transfection of si-Pontin to RBE (A) and QBC939 (B) cells. [file 6135016.f1.zip › Supplemental Table 1_BMRI_2245886.docx]

**Supplemental Table 1**. The fold of change of Pontin expression (Pontin/β-actin) in all the 16 pairs of HC tissues and matched adjacent normal bile duct tissues.

| Sample Number | | Fold Change | | Sample Number | | Fold Change | |
| --- | --- | --- | --- | --- | --- | --- | --- |
| 1 |  | 2.1 |  | 9 |  | 3.7 |  |
| 2 |  | 2.6 |  | 10 |  | 2.3 |  |
| 3 |  | 2.5 |  | 11 |  | 1.1 |  |
| 4 |  | 4.5 |  | 12 |  | 2.8 |  |
| 5 |  | 3.0 |  | 13 |  | 1.1 |  |
| 6 |  | 2.8 |  | 14 |  | 1.4 |  |
| 7 |  | 1.2 |  | 15 |  | 2.9 |  |
| 8 |  | 3.3 |  | 16 |  | 4.0 |  |
